# Differences in the Buccal Bone Marrow Distance of ≤0.8 mm in the Mandible of Patients Undergoing Sagittal Split Ramus Osteotomy among the Different Skeletal Patterns: A Retrospective Study

**DOI:** 10.3390/jcm10235644

**Published:** 2021-11-30

**Authors:** Yu-Chuan Tseng, Shih-Wei Liang, Szu-Ting Chou, Shih-Chieh Chen, Chin-Yun Pan, Chun-Ming Chen

**Affiliations:** 1 School of Dentistry, College of Dental Medicine, Kaohsiung Medical University, Kaohsiung 80756, Taiwan; yct79d@gmail.com (Y.-C.T.); ray901114@gmail.com (S.-W.L.); cherubiz@gmail.com (S.-T.C.); 2 Department of Orthodontics, Kaohsiung Medical University Hospital, Kaohsiung 80756, Taiwan; 1030414@ms.kmuh.org.tw (S.-C.C.); 910119@ms.kmuh.org.tw (C.-Y.P.); 3 Department of Oral and Maxillofacial Surgery, Kaohsiung Medical University Hospital, Kaohsiung 80756, Taiwan

**Keywords:** cone-beam computed tomography, mandibular canal, sagittal split ramus osteotomy, buccal bone marrow distance

## Abstract

This study investigated the relationship between the thickness of the ramus and skeletal patterns using cone-beam computed tomography (CBCT) for sagittal split ramus osteotomy. Ninety participants were categorized into three skeletal patterns (Class I, Class II, and Class III). The first vertical slice (slice 0) was observed in the intact mandibular canal, and then moved forward to 40 mm (slice 21) with a 2 mm interval. The thickness of buccal bone marrow (B value) was measured. A B value of ≤0.8 mm was considered to be the major risk factor causing the occurrence of postoperative lower lip paresthesia. There were 461 sides with a B value of ≤0.8 mm. There was a significant difference in the skeletal patterns [Class III (198 sides: 15.7%) > Class I (159 sides: 12.6%) > Class II (104 sides: 8.3%)]. Class II participants had significantly larger B values (2.14 to 3.76 mm) and a lower occurrence rate (≤0.8 mm) than those of Class III participants (1.5 to 3 mm) in front of the mandibular foramen (from 6 mm to 20 mm). Class III participants had significantly shorter buccal bone marrow distance and a higher occurrence rate of B values (≤0.8 mm) than Class II.

## 1. Introduction

In addition to its aesthetic implications, facial deformity is accompanied by severe malocclusion, which in turn leads to problems such as low masticatory efficiency and pronunciation difficulties. In such cases, the orthodontic treatment combined with orthognathic surgery is necessary to correct the relationship between the jaws and improve the patient’s profile. Deformities of the mandible can be divided into two types: (1) skeletal Class II malocclusion with mandibular deficiency and (2) skeletal Class III with mandibular protrusion. The type of orthognathic surgery that can be used to correct both types of mandibular development forms (mandibular deficiency or protrusion) is known as sagittal split ramus osteotomy (SSRO), and it is performed for the advancement or setback of the mandible.

The aforementioned two types of mandibular deformities differ in their structures, specifically in not only their mandible length but also their mandibular thickness. The mandibular deficiency or protrusion cannot be simply regarded as being proportionately reduced or magnified in size. Therefore, the cortical bone thickness and bone marrow thickness of the mandibular ramus could differ between the two types of mandibular deformities. Sagittal split ramus osteotomy is commonly used to correct mandibular prognathism or retrognathism. With respect to postoperative complications, the cortical bone thickness and bone marrow thickness of the mandibular ramus play an important role in the risks of bad split [1,2,3] and lower lip paresthesia [4,5,6].

Zaroni et al. [6] investigated the postoperative complications of orthognathic surgery and reported a 19.2% complication rate, including postoperative malocclusion, hemorrhage, inferior alveolar nerve injury, bad split, and infection. Möhlhenrich et al. [2] investigated the fracture patterns after bilateral sagittal split osteotomy of the mandibular ramus according to the Obwegeser/Dal Pont and Hunsuck/Epker modifications. The unfavorable and bad splits were found to be 11.3% in Obwegeser/Dal Pont method and 10% in Hunsuck/Epker modifications. Steenen and Becking [1] systematically reviewed the bad splits in bilateral sagittal split osteotomy and the incidence of bad splits was 2.3%. They found that, most frequently, fracture patterns were the buccal plate fractures of the proximal segment and lingual fractures of the distal segment. In the literature review of [1], the buccal plate was more prone to bad splits than the lingual plate. Moreover, Yamamoto et al. [7] reported that neurosensory disturbance was significantly more likely to be present 1 year after surgery, when the width of the marrow space between the mandibular canal and the external cortical bone was 0.8 mm or less.

Cone beam computerized tomography (CBCT) provides detailed images of the mandibular ramus. In present study, we aimed to analyze the buccal–lingual direction of the mandibular inferior alveolar nerve and the distance of the surrounding anatomical structures, and to investigate whether the three skeletal patterns (skeletal Class I, Class II, and Class III) were significantly different by using a CBCT study. The three skeletal classes were Class I (0° < ANB < 4°), Class II (ANB ≥ 4°), and Class III (ANB ≤ 0°). Moreover, we measured the B value, in which the buccal bone marrow thickness is the distance between the inner side of the buccal cortex and the outer side of the mandibular canal sheath. The null hypothesis was that there is no significant difference in the occurrence rate of B value (≤0.8 mm) among the skeletal patterns.

## 2. Materials and Methods

In this retrospective study, we enlisted 90 participants (CBCT: New Tom VGi evo, Imola, Italy) at the Department of Dentistry, Kaohsiung Medical University Hospital. For CBCT scan, all participants were identified with natural head position. The NNT viewer software (New Tom VGi evo, Imola, Italy) was used to view the captured images. Ninety participants were divided into three classes according to their ANB angle (A point, N: nasion, B point). The three skeletal classes were Class I (0° < ANB < 4°), Class II (ANB ≥ 4°), and Class III (ANB ≤ 0°), with 30 participants in each skeletal class. Participants with the following conditions were excluded: (1) symptoms such as craniofacial injury or tumors; (2) congenital craniofacial deformities. 

**Figure 1:**
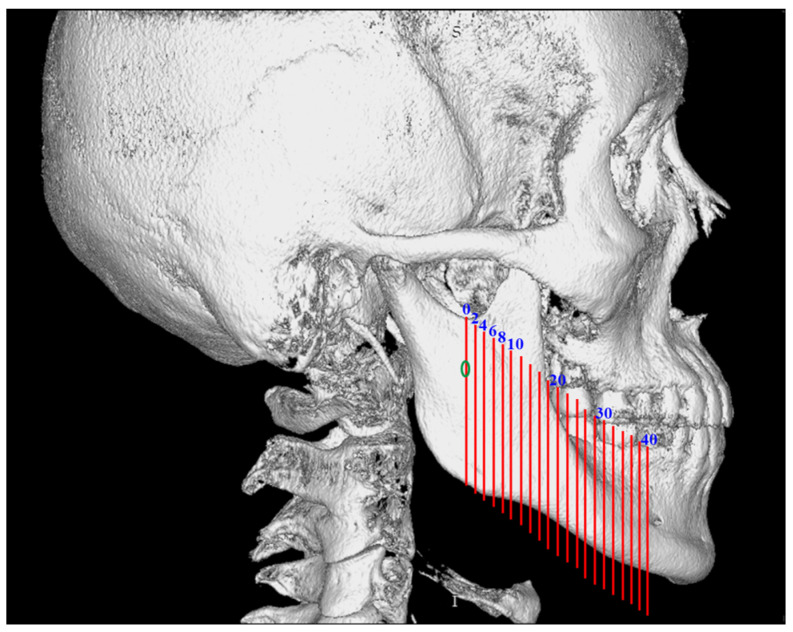
Green circle: mandibular canal (base slice: 0 mm); twenty-one vertical slices (red lines) from 0 mm forward to 40 mm.

To ensure consistency and reproducibility, the reference plane of the three-dimensional image was the FH plane (horizontal plane), which is defined as the plane constituted by the three points that pass through the right orbitale and bilateral porion. First, we demarcated the skull into the left and right sides at the orbital area of the sagittal plane. Second, we defined the horizontal section as parallel to the FH plane of the three-dimensional image. Third, we defined the coronal plane to be perpendicular to the horizontal plane and sagittal plane. We set 21 vertical slices (orthogonal projection), from which slice 0 (original slice) was used to observe the intact mandibular canal from the posterior border of ramus (Figure 1). 

**Figure 2:**
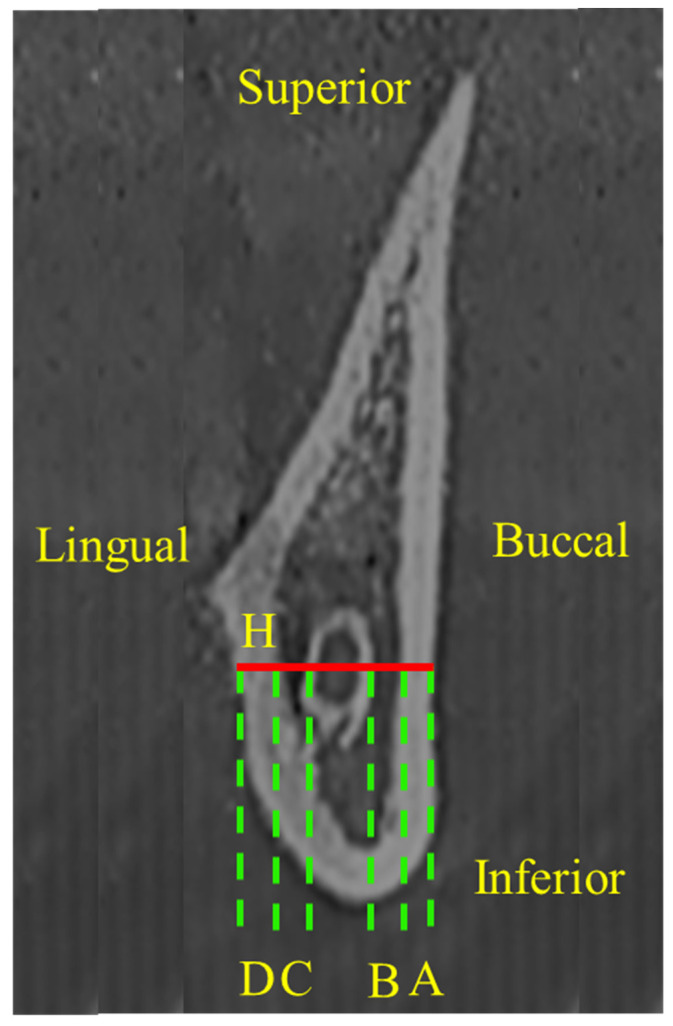
Red line distance (H: thickness of mandible). A: buccal cortical bone thickness; B: buccal cortical bone marrow thickness; C: lingual bone marrow thickness; D: lingual cortical bone thickness.

Slice 1 was located 2 mm anterior to slice 0. The region that was 2 mm in front of the previous slice was taken as a slice until the region that is 40 mm (slice 21) from the start of slice 0. Therefore, the ascending ramus was covered, which included the horizontal osteotomy line of SSRO. The thickness of cortical bones and marrows of mandibular ramus are most concerned in the procedure of SSRO. We defined a horizontal line segment (H = thickness of mandible) that passed through the center of mandibular canal; the H starts from the buccal side to lingual side of mandibular cortical bone (Figure 2). Landmarks on H were then identified, and the following line segments were defined. The thickness of cortical bones (A: buccal cortical bone; D: lingual cortical bone) and bone marrows (B, buccal cortical bone marrow: distance between the inner side of the buccal cortex and outer side of the mandibular canal sheath; C: lingual bone marrow: distance between the inner side of the lingual cortex and the outer side of the mandibular canal sheath) were measured. We also investigated the occurrence rate of B value ≤ 0.8 mm.

In this study, we conducted statistical analysis using IBM SPSS 20. Analysis of Variance (ANOVA) was used to examine the differences between the three skeletal pattern groups, and the Tukey method was used for the *post hoc* analysis, whereby a *p* value less than 0.05 was considered statistically significant. This retrospective study was reviewed and approved by the clinical trial committee of Kaohsiung Medical University Hospital (IRB No.: KMUH-IRB-20160066). 

## 3. Results

Among the 90 participants (Table 1), 30 were male and 60 were female. Among the 30 participants in the skeletal Class I group, nine were male and twenty-one were female; their mean age was 25.2 years, and their average ANB angle was 1.7°. Among the 30 participants in the skeletal Class II group, six were male participants and twenty-four were female participants; their mean age was 27.8 years, and their average ANB angle was 7.1°. Among the remaining 30 participants in the skeletal Class III group, 15 were male and 15 were female; their mean age was 22.8 years, and their average ANB angle was −4.1°. In this study, the skeletal Class III group had the youngest age in which malocclusion and poor masticatory function were identified and considered necessary to receive orthodontic treatment.

As shown in Table 2, the mean A values of participants from the three skeletal pattern groups were about from 2.5 mm to 3 mm. Significant differences (*p* = 0.022) between the A values of the skeletal groups were observed at the 10 mm anterior to the mandibular foramen. The mean A value for Class II participants (3.02 mm) was significantly larger than that for Class III participants (2.71 mm). 

**Figure 3:**
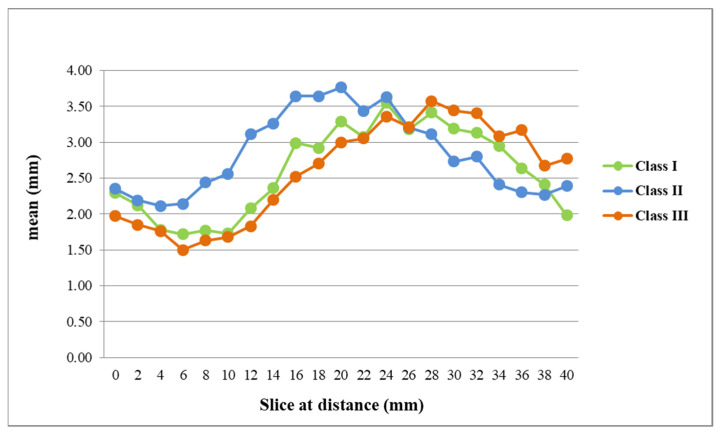
The mean B value (buccal bone marrow distance) in the three skeletal patterns.

Compared to the A value, greater differences were observed for the B value (Figure 3). Specifically, the mean B value of Class II participants (2.14 to 3.76 mm) was significantly larger than that of Class III participants (1.5 to 3 mm) at slices 3, 4, 5, 6, 7, 8, 9, and 10 (in front of mandibular foramen; from 6 mm to 20 mm). On the contrary, the mean B value of Class III participants was significantly larger than that of Class II participants at slices 15, 17, 18, and 20 (in front of mandibular foramen; from 30 mm to 40 mm). For the A + B value at slice 3, 4, 5, 6, 7, 8, and 9 (in front of the mandibular foramen; from 6 mm to 18 mm), that for Class II participants was significantly larger than that for Class III or Class I participants. On the contrary, the A + B value at slice 16, 17, and 18 for Class III participants (in front of mandibular foramen; from 32 mm to 36 mm) was significantly larger than that for Class II participants.

In Table 3, the mean C value of three skeletal patterns was <1 mm for most sections. The mean C value of Class III participants was significantly larger than that of Class II participants at slices 7, 8, 10, 11, and 13. The mean C value of Class II participants was significantly larger than that of Class III participants at slices 17, 18, and 19. The mean D value of Class III participants was significantly larger than that of Class II participants from slice 4 to slice 10. The mean D value of Class I participants was significantly larger than that of Class II participants at slices 4, 5, and 10, and Class III at slices 17, 18, and 19. The mean D value of Class II participants was significantly larger than that of Class III participants at slices 15, 17, and 18. The H value was the mandibular width; for most sections, the mean H value was from 10 mm to 13 mm. The three skeletal pattern groups also did not significantly differ in their H values for any of the sections. 

Among the 3780 sides, there were 461 sides where the B value was ≤0.8 mm (Table 4). In the comparison of sex, there was no significant difference between female (294 sided) and male (167 sides). The mean B value (≤0.8 mm) was significantly larger in female (0.33 ± 0.3 mm) than in male participants (0.25 ± 0.29 mm). In the comparison of the skeletal patterns, Class III (198 sided) was significantly greater than Class I (159 sides), whereas Class I was significantly greater than Class II (104 sides). Exploring the mean B value (≤0.8 mm), Class II (0.37 ± 0.31 mm) was significantly greater than Class III (0.25 ± 0.27 mm). Therefore, the null hypothesis was rejected. 

As shown in Table 5, the rate of occurrence of the B value (≤0.8 mm) was significantly higher in Class III participants who were at 6–20 mm anterior to the mandibular foramen than in Class II participants who were anterior to the mandibular foramen. The rate of occurrence of the B value (≤0.8 mm) was significantly higher in Class I participants at slice 4–8 than in Class II. From 4 to 12 mm anterior to the mandibular foramen, Class III had a 30 to 35% occurrence rate with a B value of ≤0.8 mm.

## 4. Discussion

The possible reasons [8,9,10,11,12] for the occurrence of postoperative nerve paresthesia include postoperative swelling, the pressure from fixing the split mandible using bone screws, and direct injury to the inferior alveolar nerve in the process of vertical osteotomy or mandibular ramus splitting. Furthermore, factors such as the patient’s age, location of vertical osteotomy, and extent of mandibular movement potentially cause the occurrence of postoperative nerve paresthesia. Cunha et al. [3] examined the influence of bone thickness on the split pattern of sagittal ramus osteotomy. They found that thinner mandibular rami were more prone to bad splits.

Kalabalik et al. [13] compared the skeletal Class I and Class III patients in the morphometric analysis of mandibular corpus relevance to sagittal split osteotomy. They concluded that prognathic mandibles had an increased risk of neurovascular complications and bad splits. In our study, the thickness of the buccal plate ranged from 2.47 mm to 3.22 mm in Class I, 2.61 mm to 3.21 mm in Class II, and 2.5 mm to 3.20 mm in Class III, respectively. In slice 5 (10 mm form mandibular foramen), the thickness of the buccal cortical plate of Class II participants (3.02 mm) was significantly larger than for Class III participants (2.71 mm). Among 21 slices, Class III had a smaller A value than Class II in 15 slices. Therefore, the risk of buccal plate fracture in class III might be higher than that of class II. Our findings were similar to the report of Kalabalik et al. [13] As for the relationship between the inferior alveolar nerve and the mandibular lingual cortical bone, all C values (width of lingual bone marrow) in the slices near the mandibular foramen were very small. At slices 7 to 13 (14 to 26 mm), the C value of the Class III group was significantly larger than that of the skeletal Class II group, except at slice 12 (24 mm). At slices 16 to 19 (32 to 38 mm), the C value of Class II was significantly larger than that of Class I or Class III. 

Lower lip paresthesia is still the main postoperative complication in SSRO. In the SSRO procedure, the inferior alveolar nerve must be protected to avoid to occurrence of lower lip paresthesia after operation. Huang and Liao [14] believed that the B value was the critical factor that caused damage to the inferior alveolar neurovascular bundle. They reported B values (ranged from 1 mm to 4 mm) from the mandibular lingula to the first molar. Our results were similar to the report of Huang and Liao [14], the mean B values ranged from 1.72 mm to 3.55 mm in Class I, 2.11 mm to 3.76 mm in Class II, and 1.5 mm to 3.44 mm in Class III, respectively. Furthermore, Huang and Liao [14] indicated that, in the sections closer to the mesial side of the mandibular foramen, the mean values of the participants with skeletal Class III were smaller than those for participants with skeletal Class II. The mean values of these two groups intersected at the second molar distal root. This meant that the Class II values were smaller than those for participants with skeletal Class III in the section anterior to the mesial root of second molar. From our findings, the participants with skeletal Class III had larger mean values than those of the participants with skeletal Class II in the slice toward the first molar direction. In the trend of mean B values, our study revealed similar results to the report by Huang and Liao [14]. Our results indicated that for the slice 3 to slice 10 (6 to 20 mm from the mandibular foramen), the mean B values of the skeletal Class II group were significantly larger than those of the skeletal Class III group. Therefore, Class II participants were likely to experience a lower incidence of nerve paresthesia than Class III after the SSRO. 

Notably, the slice moved forward from the mandibular foramen, relative to the skeletal Class III group, and the widths of the buccal bone marrow for the skeletal Class II group were larger. For the width of the lingual bone marrow, an inverse trend was noted. This indicated that, when comparing skeletal Class II and Class III groups with respect to their nerve pathways, the nerves of those in the skeletal Class III group ran along the region of mandibular foramen closer to the lingual side (i.e., smaller width of the buccal bone marrow). While moving forward, the inferior alveolar nerve of skeletal Class III participants tended to run closer to the buccal side.

Yamamoto et al. [7] reported that neurosensory disturbance occurrence in the lower lip was observed on all 10 mandibular sides (25%; 20 participates: 40 sides), in which the mandibular canal came into contact with the external cortical bone (B value = 0 mm). 

Yamamoto et al. [7] reported that females had 10 sides, and males had 6 sides with neurosensory disturbance. Furthermore, we investigated the B value (≤0.8 mm) according to sex. Regarding the occurrence rate of the sides, there was no significant difference between females (294 sides: 11.7%) and males (167 sides: 13.3%). However, males had a significantly smaller mean B value (0.25 mm) than females (B value: 0.33 mm). It seems that males may have a slightly higher occurrence of neurosensory disturbances than females.

In the study by Yamamoto et al. [7], no neurosensory disturbance was observed with a B value of ≥1.0 mm. However, neurosensory disturbance was observed on the 6 sides with a B value of ≤0.8 mm. According to the findings of Yamamoto et al. [7], a B value of ≤0.8 mm is a risk factor for neurosensory disturbance of the lower lip. In our study, there were 461 sides with a B value of ≤0.8 mm from a total of 3780 sides. Exploring the mean B value (≤0.8 mm), we found that Class III (198 sided: 15.7%) was significantly larger than Class I (159 sides: 12.6%) and Class II (104 sides: 8.3%). Class III (0.25 ± 0.27 mm) was significantly smaller than Class II (0.37 ± 0.31 mm). In the 16 of 21 slices (76.2%), Class III had a higher occurrence rate of B value (≤0.8 mm) than Class II. Moreover, we found that the rate of occurrence of the B value (≤0.8 mm) was significantly higher in Class III participants at 6 mm to 20 mm (slice 3–10) anterior to the mandibular foramen than in Class II participants. This region is the main split osteotomy path of the SSRO and located in the ramus. Therefore, we concluded that Class III participants are likely to experience a higher occurrence rate of postoperative neurosensory abnormality than Class II after the SSRO.

Huang et al. [15] studied participants with mandibular prognathism after SSRO surgery. They investigated every 2 mm from the mandibular foramen to the furcation of the mandibular first molar. Huang et al. [15] discovered that the measurement values were significantly smaller for participants with nerve paresthesia than those for participants without it at the 16, 18, 20, or 24 mm slice anterior to mandibular foramen. In this study, we compared the three skeletal pattern groups and discovered that the average values (A + B) for the skeletal Class III group were significantly smaller than those for the skeletal Class II group at the 6 mm to 18 mm region from the mandibular foramen. This region is the ascending ramus of the mandible. The width of the buccal bone marrow is of substantial clinical significance because it is closely related to whether the splitting instruments have sufficient space to operate and whether unfavorable or bad splits are likely to occur. In our findings, Class III (0.25 ± 0.27 mm) was significantly smaller than Class II (0.37 ± 0.31 mm). Integrating the results of the present study with those of Huang et al. [15], we concluded that skeletal Class III deformities feature a significantly shorter distance between the buccal side of the cortical bone and mandibular canal at the mandibular ramus than skeletal Class II deformities. Moreover, we found that the occurrence possibilities (B value ≤ 0.8 mm) of Class III (198 sided; 15.7%) and Class I (159 sides; 12.6%) were larger than Class II (104 sides; 8.3%). This short distance may result in a high probability of nerve paresthesia after SSRO surgery in the Class III and Class I patients.

## 5. Conclusions

In conclusion, our study revealed that skeletal Class III had a significantly shorter distance and higher occurrence rate of B values (≤0.8 mm) than skeletal Class II at the mandibular ramus region. Therefore, the probability of nerve paresthesia in mandibular setback for Class III was higher than the mandibular advancement for Class II after SSRO surgery. However, regarding the risk of bad split, the thickness of the bony structures is important as well as other factors, such as the curvature of the vestibular surface of the mandibular ramus.

## Figures and Tables

**Table 1:** The summary of participants’ characteristics. (*n* = 90).

Total Participant by Gender: 60 Female and 30 Male
Total participant by skeletal pattern:
30 Class I (21 female; 9 male)
30 Class II (24 female; 6 male)
30 Class III (15 female; 15 male)
Participants with buccal bone marrow distance ≤ 0.8 mm
by gender (%): 45 female (75%); 18 male (60%)
by skeletal pattern (%):
Class I (17 female, 81%; 4 male, 44%)
Class II (16 female, 67%; 4 male, 67%)
Class III (12 female, 73%; 10 male, 67%)

*n*: Number of participants.

**Table 2:** From the base plane (0 mm) to 40 mm forward, the thickness (mm, mean ± SD) of buccal cortical bone and marrow with their skeletal patterns in the One-way ANOVA with *post hoc* Tukey HSD test.

Variables		A	*p* Value	B	*p* Value	A + B	*p* Value
		(Mean ± SD, mm)		(Mean ± SD, mm)		(Mean ± SD, mm)	
0 mm	Class I	2.47 ± 0.58	0.415	2.29 ± 1.42	0.252	4.75 ± 1.43	0.129
	Class II	2.61 ± 0.68		2.35 ± 1.38		4.96 ± 1.28	
	Class III	2.50 ± 0.61		1.97 ± 1.17		4.47 ± 1.21	
2 mm	Class I	2.62 ± 0.46	0.512	2.12 ± 1.38	0.297	4.73 ± 1.43	0.201
	Class II	2.73 ± 0.66		2.19 ± 1.28		4.92 ± 1.25	
	Class III	2.65 ± 0.51		1.85 ± 1.14		4.50 ± 1.17	
4 mm	Class I	2.62 ± 0.62	0.56	1.78 ± 1.44	0.28	4.39 ± 1.44	0.115
	Class II	2.71 ± 0.73		2.11 ± 1.28		4.82 ± 1.31	
	Class III	2.57 ± 0.69		1.76 ± 1.33		4.34 ± 1.36	
6 mm	Class I	2.54 ± 0.53	0.339	1.72 ± 1.29	0.019 *	4.26 ± 1.41	0.007 *
	Class II	2.66 ± 0.60		2.14 ± 1.21	Class II > III	4.81 ± 1.35	Class II > III
	Class III	2.52 ± 0.55		1.50 ± 1.27		4.02 ± 1.37	
8 mm	Class I	2.85 ± 0.70	0.131	1.77 ± 1.40	0.003 *	4.61 ± 1.45	<0.001 *
	Class II	2.96 ± 0.70		2.44 ± 1.31	Class II > I	5.39 ± 1.45	Class II > I
	Class III	2.70 ± 0.71		1.63 ± 1.43	Class II > III	4.33 ± 1.49	Class II > III
10 mm	Class I	2.90 ± 0.53	0.022 *	1.73 ± 1.37	0.001 *	4.62 ± 1.51	<0.001 *
	Class II	3.02 ± 0.54	Class II > III	2.56 ± 1.32	Class II > I	5.58 ± 1.49	Class II > I
	Class III	2.71 ± 0.74		1.68 ± 1.42	Class II > III	4.39 ± 1.66	Class II > III
12 mm	Class I	3.10 ± 0.70	0.455	2.08 ± 1.54	<0.001 *	5.18 ± 1.64	<0.001 *
	Class II	3.08 ± 0.67		3.11 ± 1.39	Class II > I	6.19 ± 1.49	Class II > I
	Class III	2.94 ± 0.81		1.83 ± 1.60	Class II > III	4.78 ± 1.77	Class II > III
14 mm	Class I	3.12 ± 0.64	0.052	2.36 ± 1.59	<0.001 *	5.48 ± 1.59	<0.001 *
	Class II	3.12 ± 0.62		3.26 ± 1.38	Class II > I	6.32 ± 1.32	Class II > I
	Class III	2.87 ± 0.63		2.20 ± 1.65	Class II > III	5.07 ± 1.94	Class II > III
16 mm	Class I	3.11 ± 0.64	0.814	2.99 ± 1.71	0.001 *	6.1 ± 1.72	<0.001 *
	Class II	3.15 ± 0.71		3.64 ± 1.41	Class II > III	6.79 ± 1.34	Class II > III
	Class III	3.06 ± 0.81		2.52 ± 1.73		5.58 ± 1.90	
18 mm	Class I	3.22 ± 0.74	0.195	2.92 ± 1.59	0.006 *	6.14 ± 1.65	0.006 *
	Class II	3.08 ± 0.58		3.64 ± 1.33	Class II > III	6.72 ± 1.28	Class II > III
	Class III	3.01 ± 0.66		2.70 ± 1.99		5.71 ± 2.09	
20 mm	Class I	3.17 ± 0.71	0.584	3.29 ± 1.42	0.026 *	6.46 ± 1.56	0.114
	Class II	3.08 ± 0.62		3.76 ± 1.30	Class II > III	6.83 ± 1.32	
	Class III	3.20 ± 0.74		3.00 ± 1.81		6.20 ± 2.02	
22 mm	Class I	3.07 ± 0.68	0.456	3.07 ± 1.55	0.328	6.14 ± 1.58	0.157
	Class II	3.21 ± 0.59		3.43 ± 1.20		6.63 ± 1.27	
	Class III	3.09 ± 0.68		3.05 ± 1.84		6.14 ± 1.94	
24 mm	Class I	3.14 ± 0.66	0.540	3.55 ± 1.46	0.600	6.69 ± 1.61	0.737
	Class II	3.01 ± 0.64		3.63 ± 1.20		6.64 ± 1.13	
	Class III	3.11 ± 0.67		3.36 ± 1.73		6.48 ± 1.85	
26 mm	Class I	3.09 ± 0.79	0.984	3.18 ± 1.31	0.990	6.26 ± 1.45	0.996
	Class II	3.07 ± 0.46		3.20 ± 1.29		6.27 ± 1.36	
	Class III	3.07 ± 0.56		3.21 ± 1.66		6.29 ± 1.72	
28 mm	Class I	3.06 ± 0.71	0.492	3.41 ± 1.20	0.220	6.47 ± 1.43	0.153
	Class II	2.92 ± 0.60		3.11 ± 1.47		6.03 ± 1.43	
	Class III	2.96 ± 0.71		3.57 ± 1.67		6.53 ± 1.70	
30 mm	Class I	3.03 ± 0.72	0.295	3.19 ± 1.20	0.012 *	6.23 ± 1.32	0.087
	Class II	3.08 ± 0.55		2.73 ± 1.29	Class III > II	5.82 ± 1.39	
	Class III	2.91 ± 0.52		3.44 ± 1.41		6.36 ± 1.43	
32 mm	Class I	3.02 ± 0.70	0.587	3.13 ± 1.20	0.096	6.15 ± 1.38	0.047 *
	Class II	2.89 ± 0.60		2.80 ± 1.62		5.70 ± 1.55	Class III > II
	Class III	2.97 ± 0.72		3.40 ± 1.58		6.38 ± 1.54	
34 mm	Class I	2.89 ± 0.64	0.403	2.95 ± 1.17	0.017 *	5.84 ± 1.29	0.042 *
	Class II	3.03 ± 0.59		2.41 ± 1.33	Class III > II	5.43 ± 1.54	Class III > II
	Class III	3.03 ± 0.63		3.08 ± 1.35		6.11 ± 1.40	
36 mm	Class I	3.07 ± 0.73	0.607	2.64 ± 1.21	0.005 *	5.72 ± 1.36	0.020 *
	Class II	3.00 ± 0.71		2.30 ± 1.55	Class III > II	5.30 ± 1.59	Class III > II
	Class III	2.94 ± 0.75		3.17 ± 1.38		6.11 ± 1.42	
38 mm	Class I	2.85 ± 0.52	0.166	2.41 ± 1.25	0.357	5.26 ± 1.32	0.242
	Class II	3.08 ± 0.60		2.27 ± 1.35		5.35 ± 1.57	
	Class III	3.00 ± 0.62		2.67 ± 1.37		5.68 ± 1.32	
40 mm	Class I	3.17 ± 0.77	0.073	1.98 ± 1.16	0.014 *	5.15 ± 1.37	0.122
	Class II	2.77 ± 0.65		2.39 ± 1.77	Class III > I	5.15 ± 1.70	
	Class III	2.89 ± 0.81		2.77 ± 1.44		5.67 ± 1.36	

A: Buccal cortex of mandibular canal sheath; B: Dimension of mandibular canal. *: Significant, *p* < 0.05.

**Table 3:** From the base plane (0 mm) to 40 mm forward, the thickness (mm, mean ± SD) of the lingual cortical bone and marrow with their skeletal patterns in the One-way ANOVA with *post hoc* Tukey HSD test.

Variables		C	*p* Value	D	*p* Value	H	*p* Value
		(Mean ± SD, mm)		(Mean ± SD, mm)		(Mean ± SD, mm)	
0 mm	Class I	0.06 ± 0.30	0.114	1.14 ± 0.57	0.195	9.79 ± 1.61	0.641
	Class II	0.01 ± 0.06		0.97 ± 0.52		9.55 ± 1.37	
	Class III	0.00 ± 0.00		1.05 ± 0.43		9.65 ± 1.31	
2 mm	Class I	0.02 ± 0.12	0.368	1.28 ± 0.65	0.448	9.96 ± 1.50	0.735
	Class II	0.03 ± 0.18		1.15 ± 0.56		9.79 ± 1.38	
	Class III	0.00 ± 0.00		1.19 ± 0.46		9.78 ± 1.30	
4 mm	Class I	0.14 ± 0.36	0.644	1.65 ± 0.71	0.580	10.08 ± 1.60	0.480
	Class II	0.18 ± 0.42		1.59 ± 0.50		10.21 ± 1.44	
	Class III	0.11 ± 0.34		1.53 ± 0.71		9.89 ± 1.27	
6 mm	Class I	0.35 ± 0.57	0.239	1.83 ± 0.71	0.077	10.25 ± 1.66	0.116
	Class II	0.19 ± 0.42		1.56 ± 0.60		10.38 ± 1.47	
	Class III	0.29 ± 0.53		1.65 ± 0.65		9.84 ± 1.29	
8 mm	Class I	0.41 ± 0.63	0.881	2.20 ± 0.96	0.004 *	11.09 ± 1.68	0.364
	Class II	0.42 ± 0.71		1.74 ± 0.56	Class I > II	11.25 ± 1.70	
	Class III	0.46 ± 0.67		2.08 ± 0.75	Class III > II	10.84 ± 1.44	
10 mm	Class I	0.64 ± 0.80	0.255	1.87 ± 0.67	0.005 *	11.19 ± 1.78	0.140
	Class II	0.41 ± 0.70		1.58 ± 0.56	Class I > II	11.42 ± 1.64	
	Class III	0.51 ± 0.78		1.95 ± 0.70	Class III > II	10.81 ± 1.65	
12 mm	Class I	0.69 ± 0.87	0.165	2.23 ± 0.99	0.006 *	12.11 ± 1.90	0.515
	Class II	0.42 ± 0.65		1.91 ± 0.62	Class III > II	12.28 ± 1.80	
	Class III	0.65 ± 1.01		2.37 ± 0.74		11.90 ± 1.75	
14 mm	Class I	0.71 ± 0.86	0.027 *	1.88 ± 0.54	0.010 *	12.12 ± 1.84	0.392
	Class II	0.39 ± 0.70	Class III > II	1.69 ± 0.56	Class III > II	12.15 ± 1.64	
	Class III	0.83 ± 1.16		2.03 ± 0.67		11.74 ± 1.89	
16 mm	Class I	0.56 ± 0.97	0.009 *	2.20 ± 0.72	0.005 *	12.64 ± 1.73	0.879
	Class II	0.46 ± 0.77	Class III > I	1.92 ± 0.68	Class III > II	12.79 ± 1.64	
	Class III	1.02 ± 1.32	Class III > II	2.36 ± 0.82		12.76 ± 1.79	
18 mm	Class I	0.64 ± 0.73	0.010 *	1.94 ± 0.51	0.305	12.61 ± 1.72	0.905
	Class II	0.39 ± 0.73	Class III > II	1.81 ± 0.67	Class III > II	12.66 ± 1.59	
	Class III	0.91 ± 0.63		2.00 ± 0.67		12.52 ± 1.99	
20 mm	Class I	0.42 ± 0.67	0.015 *	2.35 ± 0.64	0.003 *	13.05 ± 1.72	0.370
	Class II	0.45 ± 0.71	Class III > II	1.95 ± 0.63	Class I > II	12.86 ± 1.57	
	Class III	0.83 ± 1.09		2.47 ± 1.17	Class III > II	13.33 ± 2.11	
22 mm	Class I	0.64 ± 1.01	0.007 *	2.06 ± 0.58	0.654	12.68 ± 1.71	0.424
	Class II	0.31 ± 0.57	Class III > II	2.01 ± 0.61		12.51 ± 1.65	
	Class III	0.90 ± 1.29		2.11 ± 0.71		12.95 ± 2.12	
24 mm	Class I	0.35 ± 0.55	0.054	2.39 ± 0.67	0.125	13.00 ± 1.77	0.084
	Class II	0.33 ± 0.62		2.20 ± 0.71		12.63 ± 1.55	
	Class III	0.59 ± 0.79		2.53 ± 1.15		13.39 ± 2.20	
26 mm	Class I	0.30 ± 0.60	0.006 *	2.14 ± 0.63	0.990	12.49 ± 1.78	0.267
	Class II	0.27 ± 0.52	Class III > I	2.13 ± 0.68		12.31 ± 1.52	
	Class III	0.71 ± 1.2	Class III > II	2.13 ± 0.57		12.84 ± 2.07	
28 mm	Class I	0.28 ± 0.46	0.087	2.42 ± 0.73	0.887	12.69 ± 1.91	0.173
	Class II	0.51 ± 0.88		2.35 ± 0.63		12.46 ± 1.43	
	Class III	0.57 ± 0.87		2.37 ± 0.80		13.09 ± 2.13	
30 mm	Class I	0.37 ± 0.52	0.195	2.09 ± 0.63	0.018 *	12.23 ± 1.78	0.909
	Class II	0.58 ± 0.82		2.29 ± 0.76	Class II > III	12.32 ± 1.46	
	Class III	0.61 ± 0.94		1.93 ± 0.64		12.37 ± 1.95	
32 mm	Class I	0.26 ± 0.46	0.001 *	2.38 ± 0.76	0.254	12.26 ± 1.90	0.614
	Class II	0.87 ± 1.18	Class II > I	2.46 ± 0.83		12.52 ± 1.67	
	Class III	0.58 ± 0.88		2.22 ± 0.85		12.58 ± 2.06	
34 mm	Class I	0.42 ± 0.61	0.001 *	2.22 ± 0.63	0.002 *	11.98 ± 1.61	0.338
	Class II	0.98 ± 1.04	Class II > I	2.30 ± 0.63	Class I > III	12.39 ± 1.72	
	Class III	0.52 ± 0.70	Class II > III	1.90 ± 0.61	Class II > III	11.94 ± 1.92	
36 mm	Class I	0.36 ± 0.56	<0.001 *	2.5 ± 0.73	0.004 *	11.99 ± 1.80	0.057
	Class II	1.27 ± 1.33	Class II > I	2.63 ± 0.95	Class I > III	12.85 ± 2.00	
	Class III	0.37 ± 0.57	Class II > III	2.14 ± 0.66	Class II > III	12.11 ± 1.96	
38 mm	Class I	0.53 ± 0.66	0.002 *	2.29 ± 0.68	0.004 *	11.57 ± 1.60	0.131
	Class II	1.18 ± 1.40	Class II > I	2.25 ± 0.88	Class I > III	12.44 ± 2.56	
	Class III	0.49 ± 0.69	Class II > III	1.87 ± 0.64		11.62 ± 1.94	
40 mm	Class I	0.55 ± 0.72	0.084	2.54 ± 0.79	0.089	11.79 ± 1.81	0.899
	Class II	1.06 ± 1.42		2.26 ± 1.05		12.04 ± 2.73	
	Class III	0.60 ± 0.79		2.21 ± 0.68		11.83 ± 1.97	

C: Lingual bone marrow; D: Lingual cortical bone of mandible; H: Thickness of mandible. *: Significant, *p* < 0.05.

**Table 4:** The summary of the characteristics of a total of 1890 slices (3780 sides).

Total Sides: Female (2520 Sides); Male (1260 Sides)
Buccal bone marrow distance ≤ 0.8 mm (461 sides):
by gender (female > male): female (294 sided); male (167 sides)
by skeletal pattern (Class III > Class I > Class II) *
Class I (159 sides), Class II (104 sides), Class III (198 sides)
Buccal bone marrow distance ≤ 0.8 mm (mean value: mm):
by gender (female > male) **: female: 0.33 ± 0.30 mm; male: 0.25 ± 0.29 mm
by skeletal pattern (Class II > Class III) ***
Class I: 0.31 ± 0.31 mm; Class II: 0.37 ± 0.31 mm; Class III: 0.25 ± 0.27 mm

*: Significant, *p* < 0.05 in the Bonferroni post hoc test for chi-square tests. **: Significant, *p* < 0.05 in the Student’s *t*-test. ***: Significant, *p* < 0.05 in the One-way ANOVA with post hoc Tukey HSD test.

**Table 5:** From slice 0 (0 mm) to slice 20 (40 mm), the percentage in the buccal bone marrow distance (SBM ≤ 0.8 mm) of skeletal patterns.

Variables	Class I	Class II	Class III	*p* Value	Significant		
0 mm	13.33%	15.00%	16.67%	0.877			
2 mm	18.33%	15.00%	18.33%	0.856			
4 mm	28.33%	16.67%	30.00%	0.185			
6 mm	26.67%	15.00%	35.00%	0.041 *	Class III > II		
8 mm	31.67%	15.00%	35.00%	0.031 *	Class I > II, Class III > II		
10 mm	31.67%	10.00%	30.00%	0.008 *	Class I > II, Class III > II		
12 mm	25.00%	3.33%	30.00%	0.001 *	Class I > II, Class III > II		
14 mm	21.67%	3.33%	23.33%	0.004 *	Class I > II, Class III > II		
16 mm	11.67%	1.67%	15.00%	0.034 *	Class I > II, Class III > II		
18 mm	8.33%	1.67%	18.33%	0.007 *	Class III > II		
20 mm	3.33%	1.67%	13.33%	0.016 *	Class III > II		
22 mm	3.33%	1.67%	15.00%	0.006 *			
24 mm	1.67%	0.00%	6.67%	0.069			
26 mm	1.67%	1.67%	5.00%	0.439			
28 mm	0.00%	8.33%	3.33%	0.059			
30 mm	1.67%	8.33%	1.67%	0.093			
32 mm	1.67%	11.67%	3.33%	0.038 *	Class II > I		
34 mm	1.67%	11.67%	3.33%	0.038 *	Class II > I		
36 mm	5.00%	16.67%	5.00%	0.035 *	Class II > I, Class II > III		
38 mm	11.67%	6.67%	10.00%	0.635			
40 mm	18.33%	6.67%	11.67%	0.147			

*: Significant, *p* < 0.05 in the Bonferroni post hoc test for chi-square tests.

## Data Availability

The data presented in this study are available on request from the corresponding author within the framework of a scientific cooperation.

## Abbreviations

SSRO	Sagittal Split Ramus Osteotomy
CBCT	Cone-Beam Computed Tomography
